# Successful Conservative Management of Emphysematous Cystitis With Pneumoperitoneum: A Case Report and Literature Review

**DOI:** 10.7759/cureus.43769

**Published:** 2023-08-19

**Authors:** Yurie Murata, Yuichiro Matsuo, Eiji Hiraoka

**Affiliations:** 1 Department of Internal Medicine, Tokyo Bay Urayasu Ichikawa Medical Center, Urayasu, JPN; 2 Department of Cardiology, Iwakuni Medical Center, Iwakuni, JPN; 3 Department of Clinical Epidemiology and Health Economics, The University of Tokyo, Tokyo, JPN

**Keywords:** antibiotics therapy, pneumoperitoneum, urinary tract infection, emphysematous cystitis, gross hematuria

## Abstract

Emphysematous cystitis is a rare type of urinary tract infection that is characterized by the accumulation of gas within the walls and lumen of the urinary bladder. In rarer instances, pneumoperitoneum may accompany emphysematous cystitis. When pneumoperitoneum is suspected through imaging studies in patients with emphysematous cystitis, surgical abdominal exploration is frequently performed considering the possibility of bladder perforation or coexistence of intestinal perforation. We successfully managed a case of emphysematous cystitis accompanied with pneumoperitoneum conservatively. A 90-year-old woman hospitalized with a gastric ulcer developed abrupt lower abdominal pain and hematuria. Contrast-enhanced CT revealed gas within the bladder wall, which was consistent with emphysematous cystitis, and pneumoperitoneum. No obvious bowel or bladder perforation was observed in the CT scan. Regarding her high surgical risk and clinical stability, surgical abdominal exploration was not performed, and she was managed conservatively with urethral catheter placement and antibiotics. She recovered with the treatment, and CT imaging obtained on day 18 demonstrated resolution of the bladder wall emphysema and no signs of pneumoperitoneum. We performed a literature review using MEDLINE and Japana Centra Revuo Medicina Web and confirmed 13 previously reported cases of emphysematous cystitis and pneumoperitoneum. Based on the review of these 13 cases and our case, it is difficult to predict the presence of bladder perforation solely based on peritoneal signs in physical findings or ascites on CT scans. Therefore, it would be preferable to perform surgical abdominal exploration to make a definite diagnosis and select an appropriate treatment. However, the fact that at least eight out of the 10 cases managed conservatively survived suggests that there is a specific clinical entity among patients who present with emphysematous cystitis and pneumoperitoneum that can be safely managed conservatively. Further accumulation of cases and research is necessary to determine which cases can be treated conservatively.

## Introduction

Emphysematous cystitis is a rare type of urinary tract infection (UTI) that is characterized by the accumulation of gas within the walls and lumen of the urinary bladder [1,2]. The causative organisms of emphysematous cystitis are similar to those of ordinary UTIs, with *Escherichia coli* (60%) and *Klebsiella pneumoniae* (10­-20%) being the two major organisms isolated from the urine of reported patients with this condition [2]. The exact mechanism of gas formation in emphysematous cystitis is not fully understood [1,2], but it is suggested to be related to sugar or protein fermentation by the causative microorganisms within the affected tissues [3].

Most cases of emphysematous cystitis can be treated conservatively with antibiotic infusion and bladder drainage with urethral catheter placement [2]. Surgical intervention is warranted in cases where initial conservative management has failed or in cases with suspected necrotizing cystitis or bladder perforation [4].

In rarer instances, pneumoperitoneum may accompany emphysematous cystitis [5,6]. Generally, the presence of pneumoperitoneum in radiographic tests indicates intra-abdominal emergencies that require surgical intervention, such as perforated abdominal viscus [7]. Consequently, when pneumoperitoneum is suspected through imaging studies in patients with emphysematous cystitis, surgical abdominal exploration is frequently performed considering the possibility of bladder perforation or coexistence of gastrointestinal perforation [6].

We encountered a case of emphysematous cystitis accompanied with pneumoperitoneum, which we successfully managed conservatively without performing abdominal exploration. In this report, we will present our case and review the existing literature on cases of emphysematous cystitis and pneumoperitoneum.

## Case presentation

A 90-year-old woman was hospitalized for a hemorrhagic gastric ulcer. On the day of admission, a non-contrast-enhanced CT scan was performed, which revealed no specific findings. There was no presence of gas within the bladder wall, pneumoperitoneum, or any abnormal findings in the gastrointestinal tract. The patient was managed successfully with endoscopic hemostasis and the administration of vonoprazan fumarate. From the time of her admission to the hospital until the fifth day of her stay, the patient did not report any abdominal pain. However, on the sixth day of hospitalization (day one), she developed abrupt lower abdominal pain and hematuria. Her medical history was unremarkable, with no history of diabetes mellitus. She denied smoking and alcohol use.

On physical examination, the patient was pale and experienced acute distress. Her vital signs were as follows: temperature of 38.3°C; blood pressure of 137/70 mmHg; pulse rate of 90 beats/minute; respiratory rate of 18 breaths/minute; and 93% oxygen saturation on room air. Conjunctival pallor was also noted. Although her abdomen was soft, she reported mild tenderness in her lower abdomen. There was no muscular defense or rebound tenderness. Her extremities were warm and no pneumoderma was observed. The remainder of the examinations were unremarkable.

**Figure 1:**
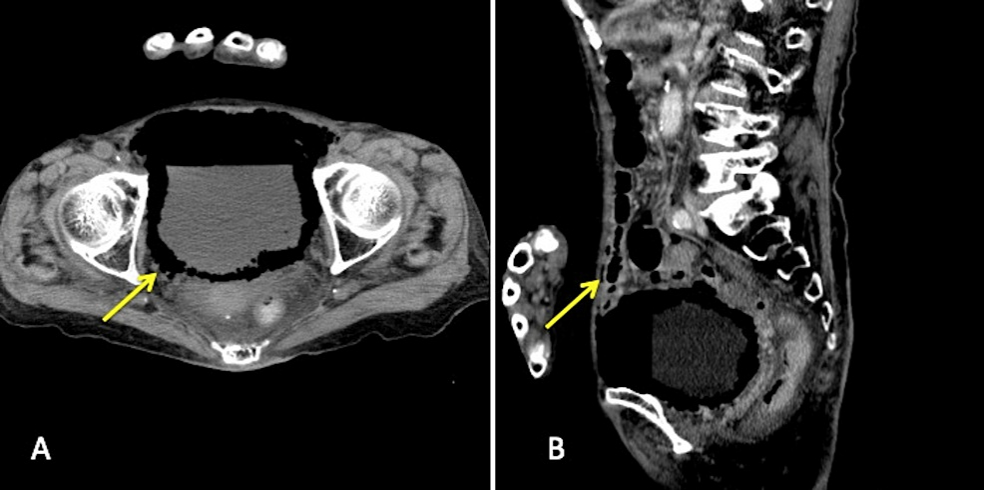
Contrast-enhanced computed tomography performed on day one A: Axial image. Multiple gas pockets are present within the bladder wall, consistent with emphysematous cystitis (arrow). No ascites was present. B: Sagittal image. Free air is present beneath the anterior abdominal wall (arrow).

Laboratory studies (Table 1) were significant for elevated levels of leukocyte count and C-reactive protein. There was no elevation in lactic acid. Gross hematuria was also observed. Subsequent contrast-enhanced CT imaging revealed novel findings that were not evident in the CT performed on the day of admission (Figure 1). These included the presence of gas within the bladder wall, indicative of emphysematous cystitis, as well as the presence of pneumoperitoneum. No unenhanced lesions or intramural gas were identified in the gastrointestinal tract, and the CT scan did not show any apparent signs of bowel or bladder perforations.

**Table 1:** Laboratory data HPF, high power field.

	Patient value on 6th day of hospitalization (day 1)	Normal value
Blood		
White blood cells (/µL)	14,300	4,000–8,000
Neutrocytes (%)	94.8	45–55
Eosinophils (%)	0	0–5
Monocytes (%)	1	45,022
Lymphocytes (%)	2.5	25–40
Hemoglobin (g/dL)	7.8	12–16
Platelets (/μL)	18.2	150,000–350,000
Total protein (g/dL)	4	6.7–8.3
Albumin (g/dL)	2.1	3.1–5.1
Aspartate aminotransferase (IU/L)	18	13–33
Alanine aminotransferase (IU/L)	18	8–42
Lactate dehydrogenase (IU/L)	175	119–229
Alkaline phosphatase (IU/L)	178	115–359
γ-glutamyl transferase (IU/L)	15	10–47
Total bilirubin (mg/dL)	0.87	0.2–1.2
Urea nitrogen (mg/dL)	22.9	8–22
Creatinine (mg/dL)	0.92	0.61–1.04
Sodium (mEq/L)	133	138–146
Potassium (mEq/L)	3.4	3.6–4.9
Chloride (mEq/L)	101	99–109
Glucose (mg/dL)	121	70–109
C-reactive protein (mg/dL)	16.71	0.0–0.29
Lactic acid (mg/dL)	7.3	4–16
Urinalysis		
Color	Red	Yellow
Appearance	Cloudy	Clear
Specific gravity	1.025	1.002–1.030
pH	5	5–8
Protein	3+	Negative
Glucose	-	Negative
Blood	3+	Negative
Urine microscopic		
White blood cells (/HPF)	>100	
Red blood cells (/HPF)	>100	

**Figure 2:**
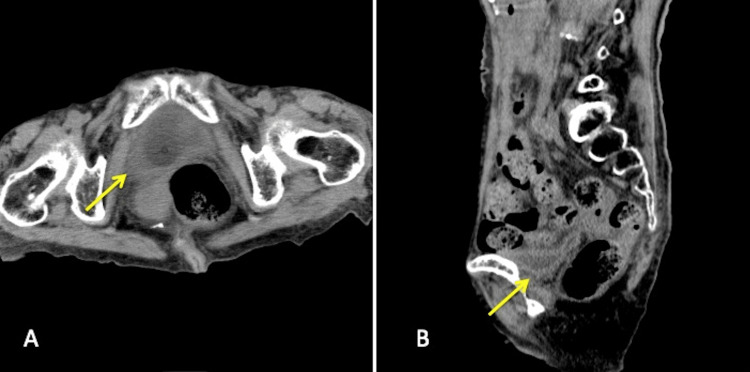
Non-contrast computed tomography performed on day 18 A: Axial image. Bladder wall emphysema was resolved (arrow). B: Sagittal image. Pneumoperitoneum was resolved (arrow).

Her clinical diagnosis was emphysematous cystitis, accompanied by pneumoperitoneum. Although we assumed that emphysematous cystitis could be conservatively managed, the possibility of concomitant bladder perforation or gastrointestinal tract perforation that might necessitate surgical intervention could not be completely ruled out. Therefore, the option of surgical abdominal exploration was considered. However, given her advanced age and malnutrition, the surgical risk was high, leading us to make the decision to treat her conservatively. A urethral catheter was placed, and intravenous treatment with meropenem was initiated. The patient remained stable, and her fever and abdominal tenderness resolved the next day. After the urine culture obtained on day one tested positive for *K. pneumoniae* on day six, antibiotic treatment was switched to ceftriaxone, which was administered for two more weeks. CT imaging obtained on day 18 demonstrated resolution of the bladder wall emphysema and no signs of pneumoperitoneum (Figure 2). She remained stable and was moved to another ward for chronic care. She was discharged from the hospital on day 40.

## Discussion

In this report, we presented a case involving an elderly frail woman who exhibited emphysematous cystitis accompanied by pneumoperitoneum. Given her overall fragile health status, we decided to manage her conservatively rather than proceeding with surgical intervention. This approach ultimately yielded successful results, and she recovered without the need for surgical intervention.

Intuitively, the presence of pneumoperitoneum in patients with emphysematous cystitis might raise suspicion of bladder perforation. The combination of bladder perforation and the presence of free air within the intraperitoneal space typically points toward intraperitoneal bladder perforation, a condition that generally necessitates surgical intervention [8]. Additionally, the possibility of gastrointestinal perforation, for which the need for surgical intervention should always be considered, should also be taken into account when pneumoperitoneum is present [9].

While there have been reported cases of bladder perforation in patients presenting with emphysematous cystitis and pneumoperitoneum that required surgical intervention [4,10], there are also cases of emphysematous cystitis and pneumoperitoneum that were successfully managed conservatively, including our case [6,11]. As there is no established standard management for this situation, we performed a literature review to describe the clinical characteristics, management, and outcomes of these patients.

We searched electronic databases (MEDLINE and Japana Centra Revuo Medicina Web) to identify published cases of emphysematous cystitis with pneumoperitoneum. On June 30, 2023, we performed a MEDLINE database search using the terms “free air,” “peritonitis,” “pneumoperitoneum,” and “cystitis,” which yielded five cases [4-6,11,12]. Additionally, we searched Japana Centra Revuo Medicina Web using the term “emphysematous cystitis (in Japanese)” and identified 145 titles, among which eight cases were diagnosed with emphysematous cystitis with pneumoperitoneum [10,13-19]. The clinical features of the 13 previously reported cases and our case are summarized in Table 2.

**Table 2:** Summary of clinical features of previously reported cases of emphysematous cystitis and pneumoperitoneum, and our case Fourteen cases of emphysematous cystitis with pneumoperitoneum were reported between 2000 and 2023, including our case.

Reference	Age	Sex	Hemodynamic status	Abdominal examination findings	Ascites	Surgical abdominal exploration	Findings	Treatment	Prognosis
Present case	90	F	Stable	Lower abdominal tenderness	Absent	Not performed	No surgical exploration performed	Conservative	Survived
Seki et al., 2000 [13]	56	F	Shock	Lower abdominal tenderness	Not reported	Not performed	Bladder perforation (apparent from imaging studies)	Conservative	Died due to a spontaneous extraperitoneal bladder rupture
Tanaka et al., 2002 [14]	80	F	Shock	Not reported	Not reported	Performed	Bladder perforation	Open surgery	Survived
Takebayashi et al., 2011 [15]	68	F	Not reported	Muscular defense	Present	Performed	No perforation	Conservative	Survived
Medina-Polo et al., 2011 [11]	65	F	Stable	No evidence of peritoneal signs	Not reported	Not performed	Not reported	Conservative	Survived
Nishino et al., 2012 [16]	82	M	Stable	No evidence of peritoneal signs	Absent	Performed	No perforation	Conservative	Survived
Tsubouchi et al., 2012 [10]	77	F	Shock	Lower abdominal tenderness	Present	Performed	Bladder perforation	Open surgery	Survived
Harada et al., 2012 [17]	59	F	Stable	Not reported	Present	Performed	No perforation	Conservative	Survived
Takano et al., 2013 [5]	83	F	Stable	Muscular defense	Present	Performed	No perforation	Conservative	Survived
Frank et al., 2016 [12]	99	F	Not reported	Not reported	Absent	Not reported	Not reported	Conservative	Not reported
Tariq et al., 2018 [6]	63	F	Stable	No evidence of peritoneal signs	Absent	Not performed	Not reported	Conservative	Survived
Hudnall et al., 2019 [4]	60	M	Tachycardia	No evidence of peritoneal signs	Absent	Performed	Bladder perforation	Open surgery	Survived
Sakai et al., 2020 [18]	80	F	Stable	Muscular defense	Absent	Performed	No perforation	Conservative	Died due to aspiration pneumonia
Cheong et al., 2021 [19]	81	M	Shock	Lower abdominal tenderness	Present	Performed	Bladder perforation	Open surgery	Survived

Out of the 14 cases, surgical abdominal exploration was performed in nine cases. Among these, four (44.4%) were found to have bladder perforation, while the remaining five cases showed no evidence of perforation. In one case, bladder perforation was apparent in imaging studies, and surgical exploration was not performed. Among the five cases with bladder perforation, four (80%) patients presented with shock, and the remaining patient presented with tachycardia accompanied by lactic acidosis, indicating that all cases with bladder perforation were hemodynamically unstable. In contrast, all five cases that were surgically confirmed to be non-perforated were hemodynamically stable. One of these five cases died (Sakai et al., 2020) due to aspiration pneumonia, but the other four survived to discharge. Peritoneal signs were positive in two out of the five (40%) perforated cases and three out of the five (60%) non-perforated cases. Ascites was observed in two out of the five (40%) perforated cases and three out of the five (60%) non-perforated cases. Among the reported cases, there were no instances of gastrointestinal perforation.

Based on the review of these 14 cases, it is difficult to predict the requirement for surgical abdominal exploration, specifically the possibility of bladder perforation or gastrointestinal perforation, solely based on peritoneal signs in physical examination findings or ascites on CT scans. Despite the observation that all of the reported patients who were hemodynamically stable did not have bladder or gastrointestinal perforation and were successfully managed conservatively (though one of these cases resulted in death due to aspiration pneumonia during the hospitalization period), hemodynamical stability alone would not be a reliable indicator of successful conservative management. It is known that cases of peritonitis due to gastrointestinal perforation can manifest with a stable hemodynamic status [9]. Therefore, it would be preferable to perform surgical abdominal exploration to make a definite diagnosis and select an appropriate treatment. However, the fact that at least eight out of the 10 cases managed conservatively survived suggests that there is a specific clinical entity among patients who present with emphysematous cystitis and pneumoperitoneum that can be safely managed conservatively.

The exact mechanism of pneumoperitoneum without bladder perforation is uncertain. It has been suggested that intramural blebs within the bladder wall may burst only at the external side of the bladder wall, causing pneumoperitoneum in the abdominal cavity without authentic transmural bladder perforation [6]. However, this hypothesis has not been well confirmed.

## Conclusions

We experienced a case involving an elderly frail woman with emphysematous cystitis accompanied by pneumoperitoneum. Due to her frailty, we opted to manage her conservatively rather than performing surgical exploration and consequently were able to successfully treat her conservatively. While it is imperative to always consider surgical abdominal exploration in patients who present with emphysematous cystitis and pneumoperitoneum to rule out the possibility of bladder or gastrointestinal perforation that requires surgical intervention, our case, along with several previous reports, suggests the existence of a distinct clinical subset of patients with emphysematous cystitis and pneumoperitoneum that can be safely managed conservatively. Further accumulation of cases and research is necessary to determine which cases can be managed conservatively.
